# *In vitro* and *in vivo* Antidiabetic Properties of Phenolic Antioxidants From *Sedum adenotrichum*

**DOI:** 10.3389/fnut.2019.00177

**Published:** 2019-11-27

**Authors:** Dil Naz, Ali Muhamad, Alam Zeb, Ismail Shah

**Affiliations:** ^1^Department of Zoology, Islamia College University, Peshawar, Pakistan; ^2^Department of Biochemistry, University of Malakand, Chakdara, Pakistan; ^3^Department of Pharmacy, Abdul Wali Khan University Mardan, Mardan, Pakistan

**Keywords:** diabetes mellitus, *Sedum adenotrichum*, glibenclamide, alloxan, α-glucosidase

## Abstract

Natural products serve as the mainstay of human life, and today, almost half of the drugs in clinical practice are the natural origin. Keeping in view the importance of medicinal plants and natural products, *Sedum adenotrichum* also known as *Rosularia adenotrichum* was selected for the present study. The crude extract of *S. adenotrichum* whole plant was obtained through a rotary evaporator. The extract was analyzed for a polyphenolic profile using high-performance liquid chromatography with a diode-array detector. The extract was subjected to detail *in vivo* antidiabetic study. In this study, body weight, blood glucose level, glycated hemoglobin, lipid profile, liver function tests, and renal function tests were performed in animal models. The extract was tested for *in vitro* α-glucosidase inhibition activity. Results of high-performance liquid chromatography with a diode-array detector chromatogram revealed a total of 22 polyphenolic compounds. No major change in body weight was noted in experimental animals. Alloxan induction led to a significant elevation in plasma glucose level. A significant decline was noted in blood glucose and glycated hemoglobin concentration in rats treated with the extract as well as with glibenclamide. Renal/liver function tests, lipid profile, alkaline phosphatase, and serum cholesterol were normalized by the extract-treated rats. The α-glucosidase inhibitory activity at 62.5 and 1,000 μg/ml was noted to be 63.97 and 80.80, respectively, both approaching to standard. The results reveal that the extract was rich in important phenolic compounds. In the antidiabetic potentials of the crude extract, there might be involved several pancreatic and extra-pancreatic mechanisms acting synergistically to induce the potent antidiabetic effect.

## Introduction

Diabetes mellitus (DM) is a global issue. It is considered the second leading cause of renal failure and blindness (1) and the seventh leading cause of death (2). Presently, 246 million people are its victim, and in the coming 20 years, the number of victims is expected over 380 million. Scientists are using various approaches to reduce their morbidity and mortality levels (3). Because of the progressive ability of the disease, precise measures and steps are necessary for control.

New drugs have been investigated for their treatment in the previous few years (4). Natural products serve as the mainstay of human life (5), and today, almost 50% of drugs in clinical practice are the natural origin. Herbal medicines are gaining more importance in diabetic complications as compared with synthetic drugs. Traditional medicines like *Acorus calamus, Aloe vera, Helianthus annuus, Bryophyllum pinnatum, Cuminum cyminum*, etc. are well-adapted in diabetic complications (6).

Natural products including animals, plants, and minerals offer a huge reservoir of active constituents. Several discoveries have explored the potential application of natural products in certain disease in different experimental models. A large number of medicinal plants are in practice for the antidiabetic purpose, and compounds with antidiabetic potentials has been isolated from medicinal plants (7). However, *Sedum adenotrichum* has never been explored for its medicinal properties including antidiabetic potential. Pakistan is rich of this natural resource. Keeping in view the importance of medicinal plants and natural products, *S. adenotrichum* also known as *Rosularia adenotrichum* was selected for the present study.

## Materials and Methods

### Plant Collection

*S. adenotrichum* whole plant was collected from Malakand. The plants were identified by Prof. Dr. Jahandar Shah, and voucher specimens Sa-74-2016 were deposited in the Herbarium. Five kilograms of the samples was originally collected. It was shade dried at room temperature, then grinded. Samples of 600 g were subjected to maceration in methanol (6 l) for 3 weeks at room temperature, followed by subjection to a rotary evaporator for complete solvent evaporation. A gummy extract was obtained, which was collected in bottles and labeled.

### Animals

This study was approved by the ethical committee of the Department of Zoology, University of Malakand. Sprague-Dawley rats (healthy, male adult, body weight = 210 ± 30 g) were obtained from the National Institute of Health, Islamabad. They were housed in the animal house of University of Malakand with free access to food and water (8). They were acclimatized prior to performance of the experiments. All the animals were provided with standard environmental conditions, food and water *ad libitum*, and light/dark cycle. Animals were divided into four groups with six rats each. All protocols of the experiment were approved by Ethical Committee via No: E-SA-11-2009 and ensured its accomplishment with provisions of the “Animal Bye-Laws 2008, Scientific Procedures Issue-I of the University of Malakand.”

### Analysis of Phenolic Compounds

Phenolic constituents were extracted from the freeze-dried sample, using water–methanol (1:9). The filtration using Agilent polytetrafluoroethylene syringe filter into high-performance liquid chromatography vials was followed. Phenolic compounds were separated and identified via Agilent Infinity Better 1260, high-performance liquid chromatography system attached with C18 reversed-phase column with a length of 100 mm, 4.6 mm width, and particle size of 3.5 μm, and diode-array detector. The separation was achieved via a solvent system comprising of solvent A (methanol–acetic acid–deionized water) (10:2:88, v/v) and solvent B (methanol–acetic acid–deionized water, 90:2:8, v/v). The separation was in 25 min of elution according to the reported method (9). Identification and quantification of the phenolic compounds were carried out against reference standards (quinic acid, p-hydroxybenzoic acid, gallic acid, salicylic acid, catechin, 3-coumaroylquinic acid, quercetin-3-galactoside, and luteolin-7-glucoside). The amount calculated was expressed as microgram per gram of the extract.

### *In vitro* α-Glucosidase Inhibition Activity

Standard procedure with little modification was adopted for examination of the α-glucosidase inhibitory potentials (10). Exactly, a 20-μl extract of various doses (62.5, 125, 250, 500, and 1,000 μg/ml) was incubated at 37°C with 50 μl of 100-mM phosphate buffer (pH 6) and 10-μl α-glucosidase (1 U/ml) in a 96-well plate for 15 min. Twenty microliters of 5-mM p-nitrophenol-glucopyranoside was added as substrate followed by additional incubation for 20 min at 37°C. The reaction was completed after the addition of 0.1 M 50 μl sodium carbonate. The absorbance was calculated for released p-nitrophenol by Multiplate Reader at 405 nm. Acarbose served as reference. The results were calculated as:

$$\begin{array}{l} \%\text{ inhibition}=\left({\text{A}}_{\text{background}}-{\text{A}}_{\text{blank}}\right)-\left({\text{A}}_{\text{sample}}-{\text{A}}_{\text{blank}}\right)\\ \;\times 100/\left({\text{A}}_{\text{background}}-{\text{A}}_{\text{sample}}\right) \end{array}$$

where *A*_*Background*_, *A*_*Blank*_, and *A*_*Sample*_ are absorbance of 100% enzyme activity (solvent plus enzyme), blank (test sample having no enzyme), and test sample (having enzyme), respectively.

### Induction of Diabetes

Single-dose alloxan of 150 mg/kg b.w. (bodyweight) intraperitoneally was used for induction of DM (11). After 2 days of alloxan administration, rats with a fasting level of plasma glucose >250 mg/dl were thought to be diabetic and used in the experiment. The crude extract was orally given continuously for 20 days (total dose 5 g/kg), whereas glibenclamide for the same period at 10 mg/kg b.w./day to the positive control. Animals were distributed among four groups (*n* = 6) as:

Group I: control, was treated with distilled water; group II: diabetic control, was treated with alloxan only; group III: alloxan-pretreated with glibenclamide served as the positive control; group IV: alloxan-pretreated with the extract.

After 7 days of alloxan injection, treatment was started through the plant extract of diabetic rats in the chronic procedure. The blood glucose was measured at 4-days intervals until the end of the experimental period. Weight and fasting glucose levels were estimated on days 1, 4, 8, 12, 16, and 20. At the end of the feeding, the rats were starved for 12 h, and weight was measured; rats were anesthetized through phenobarbitol (60 mg/kg b.w.) and slaughtered. Blood samples were collected, and serum was obtained from it. Serum biochemistry including parameters, glucose, glycated hemoglobin (HbA1c), total triglycerides, total cholesterol, alkaline phosphatase (ALP), serum urea, and serum creatinine was assayed through AMP diagnostic kits (Medizintechnik GmbH, Austria), according to manufacturer instructions.

### Biochemical Analyses

Biochemical parameters, such as glucose, HbA1c, urea, creatinine, alanine aminotransferase (ALT), ALP, cholesterol, and triglycerides were measured using methods of the respective reagent kits.

### Statistical Analysis

Data were analyzed and compared by one-way analysis of variance followed by *post-hoc* Tukey's test. *P* < 0.05 was considered to be statistically significant. Data are presented as a mean ± SEM.

## Results

### Phenolic Composition of the Extract

**Figure 1:**
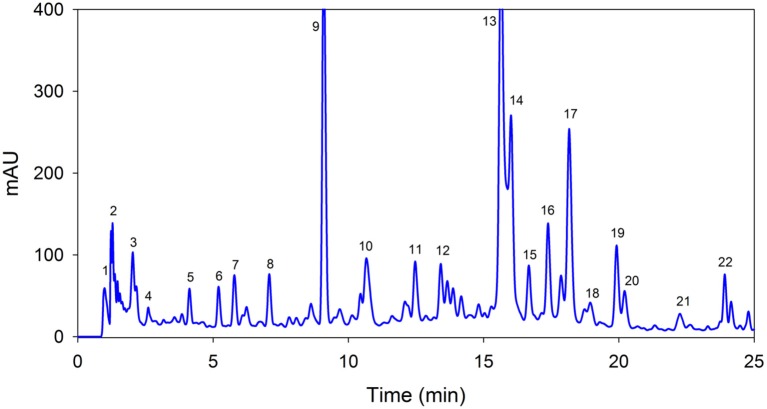
A representative high-performance liquid chromatography with a diode-array detector chromatogram of *Sedum adenotrichum* extract at 320 nm using reversed-phase C18 column. Each peak number in the chromatogram represents individual compound with characteristic details given in Table 1.

Twenty-two phenolic compounds were separated from the tested samples (Figure 1), with details as shown in Table 1. The identified compounds were quinic acid, p-hydroxybenzoic acid, gallic acid, salicylic acid, catechin, 3-*O*-coumaroylquinic acid, and naringenin at peaks 1–7, respectively. These compounds were below 100 μg/g. Peaks 8–13 were quercetin-3-galactoside, vitexin-6-malonyl-2-xyloside, luteolin-7-*O*-glucoside, apigenin-7-*O*-glucoside, apigenin-6,8-diglucoside, and kaempferol-3-*O*-sophoroside, respectively. Kaempferol-3-*O*-sophoroside (3,358.8 μg/g) was present in the highest quantity, followed by vitexin-6-malonyl-2-xyloside (2,101.1 μg/g). Quercetin-3,4-diglucoside-3-(6-feruloyl glucoside) was 1,794.8 μg/g (peak 14), quercetin-3-(p-coumaroyl-glucoside)-7-glucoside was 593.5 μg/g (peak 15), quercetin-3-(p-coumaroyl-diglucoside)-7-glucoside (797.3μg/g) was showed in peak 16, and diosmetin-7-rutinoside (1,562.2 μg/g) at peak 17. Triferuloyl-diglucoside (301.6 μg/g), kaempferol-3-(p-coumaroyl-glucoside)-7-glucoside (621.0 μg/g), kaempferol-3,7-diglucoside(352.7 μg/g), kaempferol-3-(p-coumaroyl-diglucoside)-7-glucoside (222.6 μg/g), and 4-caffeoyl-5-coumaroylquinic acid (427 μg/g) corresponding to peaks 18, 19, 20, 21, and 22, respectively.

**Table 1:** Identification and quantification of important polyphenolic compounds in *Sedum adenotrichum* extract.

**Peak**	**Rt**	**Identity**	**λmax (nm)**	**Concentration (μg/g)**
1	1.0	Quinic acid	270, 210	54.2 ± 4.1
2	1.3	p-Hydroxybenzoic acid	252	80.2 ± 3.9
3	2.0	Gallic acid	270	82.1 ± 3.6
4	2.5	Salicylic acid	302, 233	44.1 ± 4.3
5	4.1	Catechin	280	42.5 ± 2.5
6	5.2	3-*O*-Coumaroylquinic acid	314, 286	53.0 ± 4.1
7	5.8	Naringenin	282, 230	56.2 ± 3.7
8	7.1	Qurecetin-3-galactoside	356, 270	423.5 ± 13.5
9	9.1	Vitexin-6-malonyl-2-xyloside	335, 271, 232	2101.1 ± 20.2
10	10.7	Luteolin-7-glucoside	350, 256	815.1 ± 5.6
11	12.5	Apigenin-7-glucoside	336, 268	563.6 ± 4.5
12	13.4	Apigenin 6,8-diglucoside	335, 270	486.6 ± 9.5
13	15.7	Kaempferol-3-sophoroside	340, 290, 265	3358.8 ± 15.2
14	16.1	Quercetin-3,4-diglucoside-3-(6-feruloy-glucoside)	326, 267	1794.8 ± 12.3
15	16.7	Quercetin-3-(p-coumaroyl-glucoside)-7-glucoside	316, 269	593.5 ± 5.5
16	17.4	Quercetin-3-(p-coumaroyl-diglucoside)-7-glucoside	315, 263	797.3 ± 9.6
17	18.2	Diosmetin-7-rutionside	342, 264	1562.2 ± 13.6
18	18.9	Triferuloyl-dihexose	325, 264	301.6 ± 5.2
19	19.9	Kaemferol-3-(p-coumaroyl-glucoside)-7-glucoside	315, 267	621.0 ± 6.8
20	20.2	Kaempferol-3,7-diglucoside	343, 266	352.7 ± 4.5
21	22.3	Kaemferol-3-(p-coumaroyl-diglucoside)-7-glucoside	314, 264	222.6 ± 5.1
22	23.9	4-Caffeoyl-5-coumaroylquinic acid	323, 266	427.3 ± 6.3

*Values are mean of triplicate readings with a standard deviation*.

### *In vivo* Study

No significant change in body weight was noted in experimental rats as compared with control. Alloxan induction led to a highly significant elevation in plasma glucose level in all groups when compared with pre-dose levels. On day 4, a significant decline was noted in blood glucose in rats treated with the extract (*P* < 0.02), on day 8, further decrease (*P* < 0.006) was recorded, whereas on days 12, 16, and 20, a highly significant decline (*P* < 0.001) was recorded as compared with day 1 (Table 2).

**Table 2:** Plasma glucose levels of the rat groups chronically treated with *Sedum adenotrichum* (250 mg/kg b.w.) extract compared with the controls. Values are expressed as a mean ± standard error of the mean (SEM).

**Treatment days**	**Normal control**	**Diabetic control**	**Diabetic (GlTr)**	**Diabetic extract (ExtTr)**
Day 1	98.8 ± 5.3	387.3 ± 27.4	395.8 ± 25.6	411.3 ± 5.0
Day 4	108 ± 3.6	364.1 ± 21.9	308 ± 25.5	308.7 ± 9.4^*^
Day 8	106 ± 4.3	361.3 ± 25.5	251.3 ± 15.5^**^	294.0 ± 13.6^***^
Day 12	112.7 ± 5.7	373.0 ± 32.5	200.3 ± 30.6^***^	217.1 ± 25.0^***^
Day 16	103.8 ± 4.7	391.7 ± 15.3	130.5 ± 19.1^***^	155.8 ± 29.7^***^
Day 20	116.5 ± 1.3	381.0 ± 38.5	136 ± 19.1^***^	159.0 ± 21.3^***^

*Normal control (non-treated), diabetic control (alloxan treated), diabetic (ExTr): diabetic extract treated (alloxan pre-treated), diabetic (GlTr): diabetic glibenclamide treated (alloxan pretreated)*.

* *Significantly different from day 1 P < 0.05*.

** *Significantly different from day 1 P < 0.01*.

*** *Significantly different from day 1 P < 0.001*.

On day 8, a major decline (*P* < 0.01) was noted in plasma glucose levels of the glibenclamide-treated group and on 12, 16, and 20th day, a highly significant decline (*P* < 0.001) was noted as compared with day 1, whereas no significant variation was noted in normal control and diabetic control groups all over the experiment (Table 2). In the diabetic control group, plasma glucose levels remained significantly high (*P* < 0.001) as compared with the normal control, glibenclamide-treated, and extract-treated groups all over the experiment.

#### Effects on Glycated Hemoglobin

A significant (*P* < 0.001) decline was noted in the level of glycated hemoglobin in the extract-treated group compared with the diabetic control, glibenclamide-treated, and normal control groups (Table 3).

**Table 3:** Glycated hemoglobin levels, renal function tests, liver enzymes, and lipid profile of the rat groups treated with *Sedum adenotrichum* (250 mg/kg b.w.) extract compared with the controls after completion of the feeding scheme.

**Parameters (unit)**	**Normal control**	**Diabetic control**	**Diabetic (GlTr)**	**Diabetic (ExTr)**
HbA1c (%)	5.15 ± 0.2	8.62 ± 0.2	5.06 ± 0.3	5.13 ± 0.2^***^
Urea (mg/dl)	29.0 ± 1.5	143.0 ± 9.9	51.7 ± 6.5	53.1 ± 4.4^***^
Creatinine (mg/dl)	0.62 ± 0.03	1.22 ± 0.1^*^	0.60 ± 0.03	0.60 ± 0.02^***^
ALT (IU)	33.3 ± 4.3	98.0 ± 14.9	34.8 ± 1.9	52.0 ± 4.5^***^
ALP (IU)	97.8 ± 10.4	383.8 ± 21.2^**^	127.5 ± 9.8^**^	147.0 ± 3.9^***^
Cholesterol (mg/dl)	40.8 ± 2.6	88.0 ± 3.4	47.1 ± 1.7	42.8 ± 4.1^***^
Triglycerides (mg/dl)	45.5 ± 6.6	77.5 ± 5.5	56.3 ± 5.4^**^	57.1 ± 9.9^***^

*Diabetic control (alloxan treated), diabetic ExTr: diabetic extract treated (alloxan pretreated), diabetic GlTr: diabetic glibenclamide treated (alloxan pretreated)*.

* *Significantly different from control P < 0.05*.

** *Significantly different from control P < 0.01*,

*** *Significantly different from control P < 0.001. Values are expressed as a mean ± standard error of the mean (SEM)*.

#### Effects on Renal/Liver Function Tests

A very significant (*P* < 0.001) fall was recorded in the level of serum urea, ALT, and ALP in the extract-treated rats at day 20th as compared with the diabetic control, normal control, and glibenclamide-treated groups of rats (Table 3). A significant (*P* < 0.001) decline was visible in serum creatinine, in the extract-treated group at day 20th as compared with the diabetic control, normal control, and glibenclamide treated groups (Table 3).

#### Effects on Lipid Profile

A very significant (*P* < 0.001) fall was noted in serum cholesterol in the extract-treated, as compared with the diabetic control, normal control, and glibenclamide-treated groups (Table 3), whereas a significant rise was observed in serum triglyceride in the diabetic control as compared with the normal control but no significant variation as compared with the glibenclamide- and extract-treated groups (Table 3).

### *In vitro* Study

#### α-Glucosidase Inhibition Activity

As given in Table 4, the extract possesses concentration-dependent inhibitory activity for α-glucosidase. At 62.5 μg/ml, it showed 63.97 ± 1.80%, whereas acarbose (standard) showed 71.82 ± 0.7% inhibition. Similarly, at 1,000 μg/ml, a significant inhibition of 80.80 ± 1.70% occurred, almost equivalent to the standard (87.65 ± 0.8%).

**Table 4:** *In vitro* α-glucosidase inhibitory effect of extract vs. standard.

**Concentration (μg/ml)**	**Extract**	**Ascarbose (std)**
62.5	63.97 ± 1.80	71.82 ± 0.7
125	68.79 ± 1.75	74.07 ± 0.9
250	73.17 ± 1.65^*^	78.90 ± 0.5
500	77.44 ± 1.35^**^	83.05 ± 1.5
1,000	80.80 ± 1.70^**^	87.65 ± 0.8

Significantly different from controls

* P < 0.05 and

** *P < 0.01. Values are expressed as a mean ± standard error of the mean (SEM)*.

## Discussion

High-performance liquid chromatography with a diode-array detector chromatogram revealed a total of 22 polyphenolic compounds. In addition to the phenolic acids, the extract was rich in glycosides of quercetin and kaempferol. Ertaş et al. (12) quantified total glycosides of quercetin and kaempferol but could not show the exact compounds. Similarly, Oh et al. (13) identified quercetin and kaempferol glycosides in *S. sarmentosum*. These results recommend that different species of sedum are an excellent source of phenolic compounds, particularly flavonoids.

The current study was conducted to test the antidiabetic potential of a plant species *S. adenotrichum* in alloxan-induced rats, following the chronic administration of a crude extract of the plant. A significant reduction in plasma glucose concentration was observed revealing the antihyperglycemic effect of the extract. A significant reduction was noticeable in all these biochemical parameters. After chronic administration of the plant extract for 20 days, levels of plasma glucose were significantly fallen in diabetic rats. A significant decline was noted on fourth day, and a highly significant fall was noted on days 8, 12, 16, and 20, indicating that the plant possesses antidiabetic properties. Despite its application in folk medicine, proper research on a scientific basis to screen its antihyperglycemic properties has not been formerly done.

Apart from other main constituents, the plant contains flavonoids and tannins. According to literature, flavonoids and tannins possess antihyperglycemic properties (14). Significant hypoglycemic property of flavonolbioside from *Hibiscus vitifolius* flowers has been reported in scientific work (15). In this work, the antidiabetic property of the extract may be due to flavonoids and tannins, whose presence was confirmed in the preliminary phytochemical testing. The possible hypoglycemic mechanism of the plant was probably through augmentation of beta cells insulin discharge or because of improved carrying of blood glucose peripherally.

Several mechanisms are thought to work synergistically in diabetic animals for lowering plasma glucose levels. Some plants possess a property similar to oral antihyperglycemics like sulfonylureas, which decrease the level of blood glucose in normoglycemic animals (16), whereas several antihyperglycemics show no effect on levels of plasma glucose in normal state and show the effect on diabetic animals only like biguanides, such as metformin (17). Most probably, this plant may possess metformin-like mechanism as in trials; the plant extract showed no effect on the level of plasma glucose in normoglycemic rats.

Too much rise in the level of plasma glucose during DM affects plasma proteins. Moieties of glucose react with hemoglobin forming HbA1c, and amount of raise in HbA1c is thought to be directly proportional to the rise in the level of fasting blood glucose (18). Therefore, HbA1c works as a marker for estimation of status of diabetes. HbA1c is formed increasingly and permanently for a time and is stable over the lifespan of red blood cells. It is not affected by diet, exercise, or insulin and even on test day. Since it is slowly formed and dissociates hardly, it shows an actual level of blood glucose (19). Hence, HbA1c is thought to be a trustworthy index and is an imperative parameter in prognosis and management of the disease (20). In this work, the extract administration for 20 days leads to a significant fall in the level of HbA1c in diabetic rats (21). This effect of the extract on level of HbA1c is likely to be because of enhancement in the secretion of insulin leading to plasma glucose decline; a fall in plasma glycosylated hemoglobin is directly proportional to a fall in plasma glucose (22).

In this work, a significant rise was noted in the level of serum urea and serum creatinine in diabetic rats demonstrating renal failure. Raised urea levels in diabetic rats were probably because of raised catabolism of protein or deamination of amino acid for gluconeogenesis (23). Diabetic rats' exposure to a chronic dose of the extract induced a significant fall in creatinine and serum urea, thus improving renal function. Similar observations have been reported for the extract of other plant species (24). The improvement in renal function with the extract in this work maybe because of its antidiabetic property, leading to rising of distorted metabolic status in animals and by the regenerative capability of renal tubules (25).

Liver function is determined through liver enzymes. ALT is contained in cells of the liver, kidney, and muscles, while ALP is present in the intestine, bones, liver, and placenta (26). ALT is involved in catalysis of alanine to pyruvate and glutamate, acting as an indicator for hepatic injury detection. Aminotransferases and alkaline phosphatases are present in the cytoplasm and are released after damage to cells and act as an index for functional integrity loss of cell membrane (27). Level of serum ALP represents the hepatocellular function. A rise in the level of serum ALP might be due to enhanced enzymes formation (28). During hepatocellular injury and parenchymal cell necrosis, these enzymes are discharged from damaged tissue to the bloodstream (29).

Alloxan leads to a rise in the level of serum ALP and ALT in diabetic rats, while restoration in the level of ALT, ALP in extract, and glibenclamide-treated groups. Hepatic damage is frequently observed due to the high concentration of these enzymes present in the liver. Successful control on ALP due to the extract indicates early improvement in secretary phenomena of liver cells indicating the hepato-protective property of the extract. This hepato-protective property of the extract might have occurred because of flavonoids' presence, which possesses free radical scavenging properties (30). An elevated level of these enzymes shows hepatic injury since these are thought to be the main indicator of normal hepatic function. Restoration to normal level shows recovery from damage.

According to several scientific reports, hyperglycemia is accompanied by hyperlipidemia in diabetes (31). Elevated cholesterol level is the main risk factor in coronary artery disease (32), which is a leading reason of morbidity in diabetic rats (33). Triglycerides and cholesterol level were raised in the diabetic control group, which is linked to a significant alteration in metabolism and structure of lipid (34). Abnormalities in cellular metabolism of cholesterol are responsible for elevated changes in cholesterol in diabetes; the authentic mechanism causing these alterations has remained to be determined (35).

The significant elevation observed in triglycerides was most probably due to plasma insulin shortage in diabetic rats. During ordinary circumstances, insulin potentiates lipoprotein lipase and succeeding hydrolysis of triglycerides (36). Most probably, the lowering level of triglyceride in diabetic rats is because of a raised level of plasma insulin. Several plants species have been tested positive to reduce triglycerides, as diabetes leads to elevated triglycerides (37). In this work, triglycerides and cholesterol were significantly elevated. After treatment with the plant extract, a significant fall in triglycerides and cholesterol was observed. Thus, the extract showed hypoglycemic and hypolipidemic potentials. Though the level of insulin was not assessed in this work, probably, the lack of insulin in the condition of diabetes is the cause of elevation in triglycerides and cholesterol (36).

The results of this work show the antidiabetic property of the crude extract in diabetes-induced rats. Many pancreatic and extra-pancreatic mechanisms may be involved in acting synergistically to induce potent antidiabetic property. This work shows that glibenclamide and the extract possibly adopt a similar mechanism to produce the hypoglycemic effect in rats. This work also suggests that the extract is valuable in controlling biochemical parameters connected with DM, such as glycosylated hemoglobin, creatinine, serum urea, triglycerides, cholesterol, and also properties of liver enzymes like ALP and ALT.

## Conclusion

This work shows scientific confirmation that the use of *S. adenotrichum* that is rich in important phenolic compounds can be used as a therapeutic source for diabetes. It is suggested to carry out long-term research work to recognize and isolate the active moiety responsible for antidiabetic property and to expose the real mechanism involved in glucose- and lipid-lowering properties of the plant.

## Data Availability Statement

The datasets generated for this study are available on request to the corresponding author.

## Ethics Statement

The animal study was reviewed and approved by the ethical committee of the Department of Zoology, University of Malakand.

## Author Contributions

AZ: conceptualization. IS: data curtain. DN: formal analysis. AM: funding acquisition. DN and AZ: investigation. AZ: methodology. AM: project administration. IS: resources. AM: supervision. IS: validation. DN: writing—original draft. AZ: writing—review and editing, revision.

### Conflict of Interest

The authors declare that the research was conducted in the absence of any commercial or financial relationships that could be construed as a potential conflict of interest.
